# Stable Domain Assembly of a Monomolecular DNA Quadruplex: Implications for DNA-Based Nanoswitches

**DOI:** 10.1016/j.bpj.2016.04.031

**Published:** 2016-05-24

**Authors:** Besik Kankia, David Gvarjaladze, Adam Rabe, Levan Lomidze, Nunu Metreveli, Karin Musier-Forsyth

**Affiliations:** 1 Department of Chemistry and Biochemistry, The Ohio State University, Columbus, Ohio; 2 Institute of Biophysics, Ilia State University, Tbilisi, Republic of Georgia

## Abstract

In the presence of K^+^ ions, the 5′-GGGTGGGTGGGTGGG-3′ (G3T) sequence folds into a monomolecular quadruplex with unusually high thermal stability and unique optical properties. In this study we report that although single G3T molecules unfold and fold rapidly with overlapping melting and refolding curves, G3T multimers (G3T units covalently attached to each other) demonstrate highly reproducible hysteretic behavior. We demonstrate that this behavior necessitates full-length tandem G3T monomers directly conjugated to each other. Any modification of the tandem sequences eliminates the hysteresis. The experimentally measured kinetic parameters and equilibrium transition profiles suggest a highly specific two-state transition in which the folding and unfolding of the first G3T monomer is rate-limiting for both annealing and melting processes. The highly reproducible hysteretic behavior of G3T multimers has the potential to be used in the design of heat-stimulated DNA switches or transistors.

## Introduction

Nucleic acid quadruplexes, discovered more than 50 years ago (1), are currently experiencing a renaissance because of their 1) potential role in the regulation of gene expression (2, 3, 4, 5), 2) widespread occurrence in aptamers (6, 7, 8, 9), and 3) potential in biotechnological applications. The later includes their use as DNA nanowires (10, 11), switches (12, 13, 14, 15, 16), driving forces of endergonic reactions (17), detection probes (18, 19), and high-affinity DNA couplers (20).

**Figure 1:**
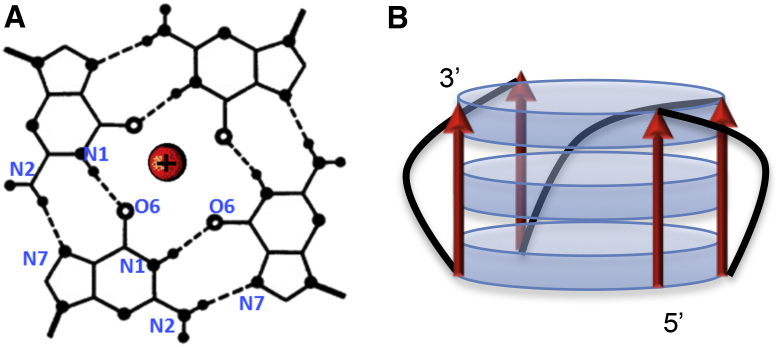
(*A*) G-quartet with cation (*red*) in the center; and (*B*) schematic representation of G3T quadruplex with all parallel G-tracts (*red*) and chain-reversal T-loops (*black*). Blue discs represent G-quartets. To see this figure in color, go online.

The main structural element of quadruplexes is the guanine (G)-quartet, which is formed by four Gs associated through Hoogsteen hydrogen bonds and stabilized by coordination bonds to centrally located cations (Fig. 1 *A*). Due to the cation coordination and stacking interactions, the quadruplexes are extremely stable. This is particularly true for the monomolecular DNA quadruplex GGGTGGGTGGGTGGG (G3T), which in the presence of 1 mM K^+^ melts at 75°C (21). The remarkable stability of the G3T quadruplex is attributed to the all-parallel alignment of GGG-tracts and chain-reversal single-loops (Fig. 1 *B*) (21, 22). This tertiary fold represents the most stable structural motif, since any modification (i.e., elongation or shortening G-tracts, nucleotide exchanges, loop elongation) significantly destabilizes it (21, 23, 24, 25, 26).

Recently, we described the intermolecular tetrahelical architecture of DNA that employs G3T quadruplexes as building blocks that form uninterrupted higher-order assemblies (27). The oligo(G3T) assemblies unfold as a single cooperative entity at significantly higher temperatures than that of the G3T monomer. However, the reason for this dramatic increase in thermal stability was not explored. We hypothesize that domain-domain interactions are responsible for the increased stability of higher-order G3T assemblies, which may be revealed by differences between the melting and refolding profiles, i.e., hysteresis. Hysteresis is well documented for DNA polymers and multimolecular assemblies (i.e., DNA nanostructures or intermolecular quadruplexes) and explained by kinetic barriers to the nucleation process (28, 29, 30, 31). Short monomolecular constructs usually fold rapidly; however, hysteretic behavior has been reported for monomolecular triplexes (32), i-motifs (33), and DNA quadruplexes (28, 29, 34, 35). In all these cases, kinetics were very slow (i.e., days) preventing direct and reproducible measurements of the kinetic parameters and equilibrium transition profiles.

In this study, we investigate the contribution of G3T-G3T (domain-domain) interactions in the energetics of oligo(G3T) assemblies. The thermal unfolding studies indeed reveal highly reproducible hysteretic loops for oligo(G3T) constructs and the central role of G3T domain interactions in this behavior. The data suggest that the hysteretic behavior of oligo(G3T) can be used to program heat-stimulated structural rearrangements in DNA (i.e., DNA switches or transistors).

## Materials and Methods

The majority of DNA oligonucleotides, obtained from Integrated DNA Technologies (Coralville, IA), were polyacrylamide gel electrophoresis-purified unless otherwise noted. The concentration of the DNA oligonucleotides was determined by measuring ultraviolet (UV) absorption at 260 nm as described earlier (36). All measurements are performed in a buffer solution consisting of 0.1 mM KCl, 10 mM Tris-HCl at pH 8.7.

Oligonucleotide names and sequences from 5′ to 3′:

**G3T**, GGGTGGGTGGGTGGG; **(G3T)**_**2**_, GGGTGGGTGGGTGGGGGGTGGGTGGGTGGG; **(G3T)**_**1.25**_**R**, GGGTGGGTGGGTGGGGGG; **(G3T)**_**1.25**_**L**, GGGGGGTGGGTGGGTGGG; **(G3T)**_**1.5**_**R**, GGGTGGGTGGGTGGGGGGTGGG; **(G3T)**_**1.5**_**L**, GGGTGGGGGGTGGGTGGGTGGG; **(G3T)**_**1.75**_**R**, GGGTGGGTGGGTGGGGGGTGGGTGGG; **(G3T)**_**1.75**_**L**, GGGTGGGTGGGGGGTGGGTGGGTGGG; **(G3T)**_**2**_**(G1-)**, GGTGGGTGGGTGGGGGGTGGGTGGGTGGG; **(G3T)**_**2**_**(G30-)**, GGGTGGGTGGGTGGGGGGTGGGTGGGTGG; **(G3T)-T-(G3T)**, GGGTGGGTGGGTGGGTGGGTGGGTGGGTGGG; **(G3T)-T**_**3**_**-(G3T)**, GGGTGGGTGGGTGGGTTTGGGTGGGTGGGTGGG; **(G3T)-T**_**5**_**-(G3T)**, GGGTGGG-TGGGTGGGTTTTTGGGTGGGTGGGTGGG; **(G3T)**_**3**_, GGGTGGGTGGGTGGGGGGTGGGTGGGTGGGGGGTGGGTGGGTGGG; **(G3T)**_**4**_, GGGTGGGTGGGTGGGGGGTGGGTGGGTGGGGGGTGGGTGGGTGGGGGGTGGGTGGGTGGG; **(G3T)**_**3**_**(G17→T)**, GGGTGGGTGGGTGGGGTGTGGGTGGGTGGGGGGTGGGTGGGTGGG; **(G3T)**_**3**_**(G2→T)**, GTGTGGGTGGGTGGGGGGTGGGTGGGTGGGGGGTGGGTGGGTGGG.

Circular dichroism (CD) spectra were obtained with a Jasco-815 spectropolarimeter (Easton, MD) using 2–8 *μ*M oligonucleotide solutions in 1 cm cells. Isothermal and temperature-dependent UV measurements were performed using a Varian UV–visible spectrophotometer (Cary 100 Bio, Santa Clara, CA) at 295 nm. The optical devices were equipped with thermoelectrically controlled cell holders. In a typical experiment, oligonucleotide samples were mixed and diluted into the desired buffers in optical cuvettes. The solutions were incubated at 100°C for 2 min in the cell holder before ramping to the desired temperature. The slowest temperature gradient, 0.02°C/min, was realized by changing the temperature in 2°C steps and waiting 100 min at each point. In isothermal folding experiments, the quadruplexes were fully unfolded by incubating at 100°C for 2 min, inserted in the cell holder at desired temperature, and kinetic measurements were immediately initiated. Similarly, the unfolding kinetics were monitored in a fully folded quadruplex by incubating at 45°C for 5 min. An Arrhenius plot, ln(*k*) versus 1/*T*, was constructed using these kinetic data, and activation barriers ere estimated from the slopes.

## Results and Discussion

### (G3T)_2_ quadruplex reveals unusually large thermal hysteresis

**Figure 2:**
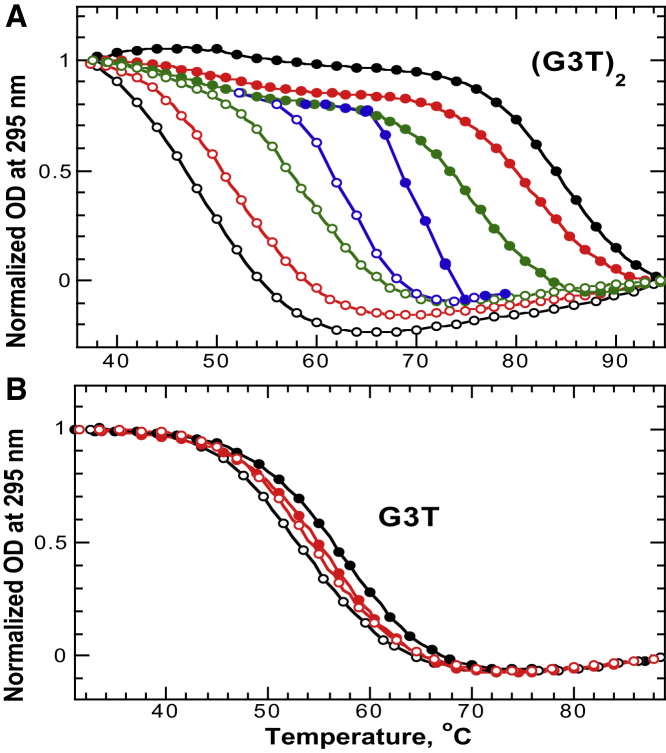
UV unfolding and refolding experiments of (*A*) (G3T)_2_ and (*B*) G3T in 0.1 mM KCl. Color code for the temperature gradient: 1°C/min (*black*), 0.5°C/min (*red*), 0.1°C/min (*green*), and 0.02°C/min (*blue*). Solid and open circles correspond to unfolding and refolding curves, respectively. To see this figure in color, go online.

UV unfolding/refolding curves of a G3T dimer, (G3T)_2_, recorded at a 1°C/min temperature gradient, demonstrate ∼35°C hysteresis (Fig. 2 *A*). Under the same experimental conditions, G3T monomer shows insignificant hysteresis, ∼3°C, which is typical for G3T quadruplexes and completely disappears at a 0.5°C/min heating rate (Fig. 2 *B*) (24, 37). The (G3T)_2_ hysteresis decreases with the temperature gradient: at 0.5°C/min and 0.1°C/min rates it drops to ∼29°C and ∼16°C, respectively. However, it does not disappear completely even at a 0.02°C/min heating/cooling rate (Fig. 2 *A*). To test whether the hysteresis of (G3T)_2_ is due to low concentration of KCl (0.1 mM), we performed similar melting studies in the presence of 0.2 and 0.5 mM KCl. The profiles revealed similar hysteresis loops but shifted to ∼8°C and ∼15°C higher temperatures, respectively (Fig. S1 in the Supporting Material).

**Figure 3:**
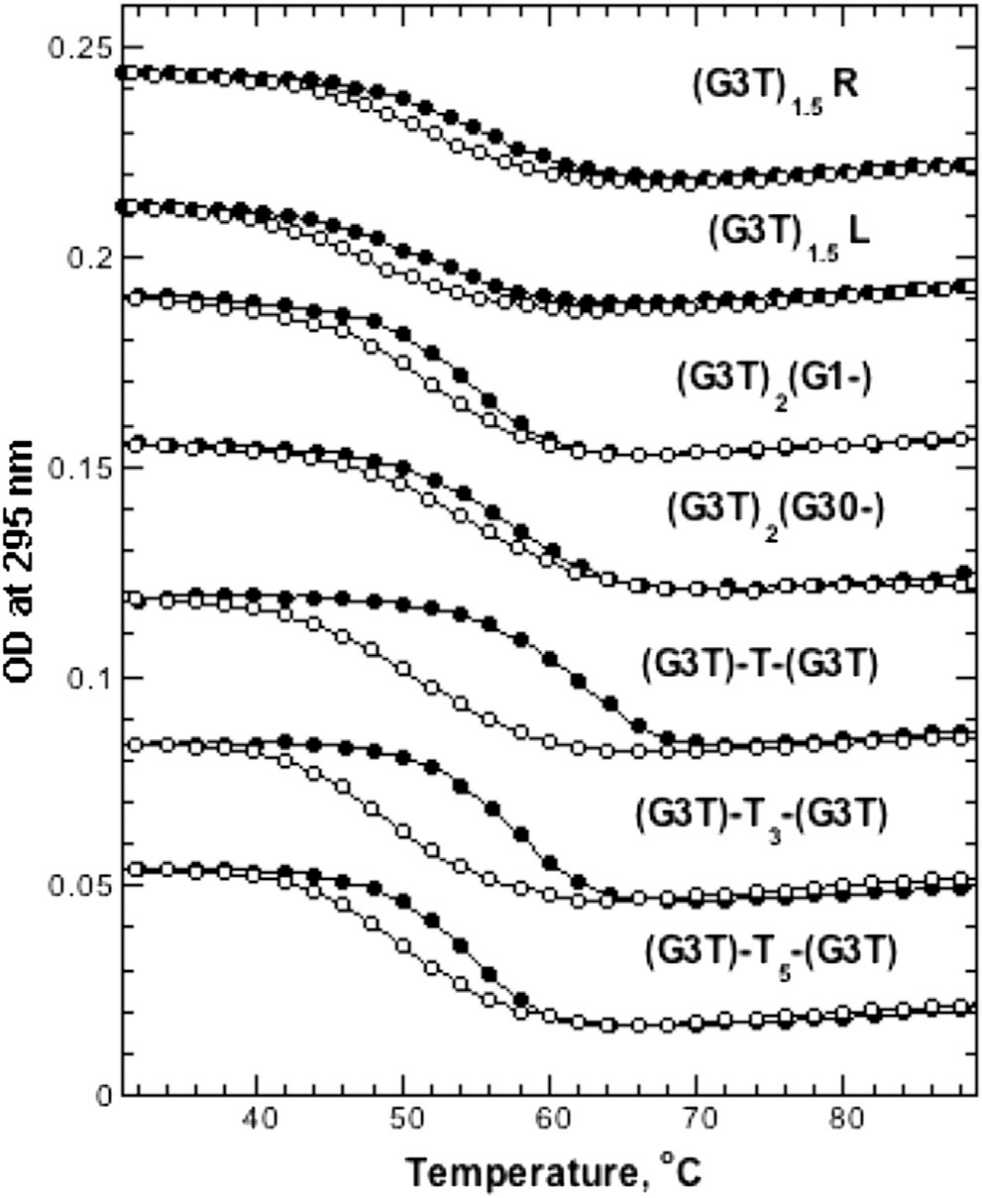
UV unfolding and refolding experiments of (G3T)_2_ variants measured using a 1°C/min temperature gradient in 0.1 mM KCl. Upper four curves correspond to truncations and lower three curves correspond to T-insertions. Curves are offset for clarity.

**Figure 4:**
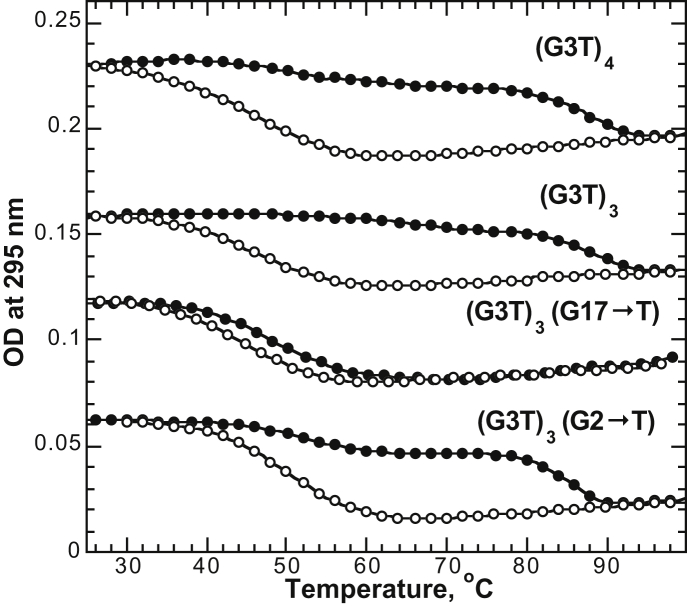
UV unfolding and refolding experiments of (G3T)_3_, (G3T)_4_, and variants measured using a 1°C/min temperature gradient in 0.1 mM KCl. Curves are offset for clarity.

The melting curves of (G3T)_2_ reveal a small transition around 52°C, which we attribute to the presence of a small fraction of failure sequences due to the less than 100% coupling efficiency of chemical synthesis. Because G3T quadruplex formation can be inhibited by even a single-nucleotide deletion (see Figs. 3 and 4) (21), truncated (G3T)_2_ sequences will form one G3T quadruplex with a flapping tail instead of the desired adjacent two G3T quadruplexes. For instance, a (G3T)_2_ sequence missing the 5′-terminal guanine would form a single G3T quadruplex with a GGTGGGTGGGTGGG tail at the 5′-end. The nonquadruplex extensions have a destabilization effect on the single G3T quadruplex (38), which explains the ∼3°C reduction in the melting transition relative to an isolated quadruplex (*T*_m_ = 55°C) (27).

### Any truncation of (G3T)_2_ eliminates the hysteresis

To determine the effect of truncation on the hysteresis observed for intact (G3T)_2_, we performed a series of melting experiments using a G3T monomer extended by addition of GGG, GGGTGGG, and GGGTGGGTGGG segments at the 3′- or 5′-end forming (G3T)_1.25_, (G3T)_1.5_, and (G3T)_1.75_ constructs (see Materials and Methods). All of these constructs unfold at ∼53°C without significant hysteresis as shown for (G3T)_1.5_L and (G3T)_1.5_R constructs in Fig. 3. Thus, truncation of (G3T)_2_ results in elimination of the hysteresis. These experiments clearly indicate that two intact G3T units are essential for the hysteretic behavior. Moreover, deletion of even a single guanine from either end of (G3T)_2_ also eliminated the hysteresis (Fig. 3, (G3T)_2_(G1-) and (G3T)_2_(G30-)).

### Insertion of T-linkers between G3T units strongly affects the hysteresis

The insertion of even a single nucleotide between G3T units reduces (G3T)_2_ hysteresis by ∼70%, and constructs with T_3_ and T_5_ linkers revealed only ∼7°C and ∼3°C deviations, respectively. The latter is typical for G3T monomer at a 1°C/min heating/cooling rate (Figs. 2 and 3). Thus, the hysteretic behavior is extremely sensitive to the separation between G3T monomers.

Three important features can be derived from the UV melting studies shown in Figs. 2 and 3. First, the hysteretic loops are highly reproducible (see Fig. S2), which suggests that the hysteresis is caused by specific and reproducible phase transitions. Second, decrease in temperature gradient is accompanied by shifting both heating and cooling curves indicating on both slow unfolding and folding processes. Third, the hysteretic behavior necessitates two full-length G3T monomers directly conjugated to each other.

### (G3T)_3_ and (G3T)_4_ also reveal large hysteresis

To determine if the hysteretic behavior observed for (G3T)_2_ is also present in the longer G3T multimers, we conducted melting experiments of a trimer, (G3T)_3_, and a tetramer, (G3T)_4_. These measurements revealed similar hysteresis loops with slightly higher unfolding temperatures of ∼88°C (Fig. 4). Thus, the hysteretic behavior is characteristic of G3T multimers in general.

Based on the lack of hysteresis observed for the (G3T)_2_ truncations and linker insertions (Fig. 3), we hypothesized that similar modifications to higher-order polymers will display variable effects depending on the location of the defect. Indeed, as shown in Fig. 4, a G17→T substitution in the middle G3T monomer converts this monomer into a nonspecific linker. As a result, the terminal G3T quadruplexes melt at ∼55°C with little hysteresis similar to G3T-T_5_-G3T. In contrast, the same G→T exchange in the terminal G3T units (i.e., second position of (G3T)_3_, see G2→T construct in Fig. 4) does not significantly affect the hysteresis loop since this construct still forms a (G3T)_2_ quadruplex dimer at the 3′-end. These data further support the conclusion that hysteretic behavior requires intact and directly conjugated G3T quadruplexes. Moreover, this is true not only for G3T dimers, but for higher-order polymers (i.e., (G3T)_3_).

### Hysteresis is attributed to a monomolecular structure with all-parallel quadruplexes

Melting experiments performed at 1, 3, and 10 *μ*M (G3T)_2_ (Fig. S3) resulted in essentially the same folding/unfolding profiles. Isothermal mixing of K^+^ ions with (G3T)_3_ and (G3T)_4_ at different strand concentrations also did not show any concentration dependence (27). The concentration independence of thermal stability and kinetic parameters is a clear indication that the system is monomolecular.

Under different buffer conditions, the CD spectra of G3T and its variants (including multimers) show a positive signal at 260 nm and a negative signal at 240 nm (27, 38). This is typical for all-parallel G-quartets with exclusively *anti*-glycosyl bonds (39, 40) formed by chain-reversal single-nucleotide loops (21, 41). Antiparallel quadruplexes demonstrate completely different CD spectra: positive bands with maxima at ∼245 and ∼295 nm and a negative peak at ∼265 nm (39, 42, 43). Thus, if the hysteresis is caused by structural rearrangements between antiparallel and parallel conformations, it should be detected by CD measurements. Our (G3T)_2_ samples, in the presence of 0.1 mM KCl, prepared by rapid cooling and slow annealing, revealed exactly same CD profiles corresponding to all-parallel quadruplexes (Fig. S4).

The optical studies support the monomolecular nature of the hysteresis and exclude antiparallel-parallel rearrangements as a cause for the slow kinetics.

### Isothermal experiments suggest fully reversible two-state transition

**Figure 5:**
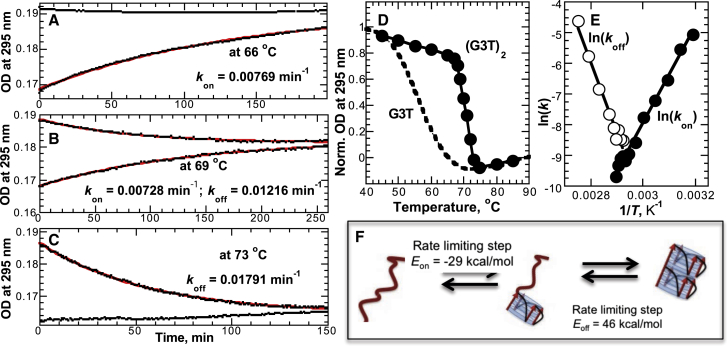
Isothermal folding and unfolding of (G3T)_2_ in 0.1 mM KCl. (*A*–*C*) Representative kinetics of simultaneous folding and unfolding at different temperatures are shown. At 66°C we observe only folding; at 73°C, only unfolding; and at 69°C, both processes are visible. The red lines correspond to a single-exponential fit of the data. (*D*) The reversible melting curve of (G3T)_2_ constructed from isothermal experiments (*solid circles*) is shown. The reversible melting of G3T from Fig. 2*B* (*dashed line*) is shown for clarity. (*E*) Arrhenius plot to determine activation energies for folding (*solid circles*) and unfolding (*open circles*) of (G3T)_2_ is shown. (*F*) Suggested model describing the two-state phase transition is shown. To see this figure in color, go online.

As shown in Fig. 2 *A*, (G3T)_2_ hysteresis correlates with the temperature gradient: the slower the gradient, the smaller the hysteresis. However, at the slowest temperature gradient used in the melting experiments, 0.02°C/min, the hysteresis is still significant, ∼8°C. Recording the melting experiments with slower temperature gradient was not practical. We next carried out isothermal folding/unfolding experiments, which in addition to characteristic equilibrium melting behavior provide kinetic parameters. To simultaneously monitor both folding and unfolding, two identical cuvettes were filled with the same (G3T)_2_ solution. One of the cuvettes was incubated for 2 min at 100°C (to unfold the quadruplex) and another was incubated for 5 min at 45°C (to ensure that the quadruplex was fully folded). The isothermal folding/unfolding experiments were initiated by simultaneously inserting both cuvettes into the UV cell holder equilibrated at the desired temperature (see Fig. 5, *A*–*C*). These isothermal measurements revealed that (G3T)_2_ unfolding is a fully reversible process characterized by a highly cooperative transition with a *T*_m_ of 69°C (Fig. 5 *D*). At 66°C, the folding time is ∼130 min (Fig. 5 *A*) and the process accelerates with a decrease in temperature. For example, the folding time is ∼5 min at 45°C (data not shown). Similarly, above 85°C we observed rapid unfolding (less than 5 min, data not shown) whereas it slows down at lower temperatures (e.g., 56 min at 73°C, Fig. 5 *C*).

An Arrhenius plot revealed a linear dependence for both folding and unfolding processes with the intercept at 69°C corresponding to the *T*_m_ of the transition (Fig. 5 *E*). This is indicative of a two-state transition. The activation energy of the folding is negative, which suggests that the reaction accelerates at lower temperatures. This is typical for DNA structures and suggests the nucleation zipper mechanism for the folding process (44). Interestingly, the *E*_on_ value, −29 kcal/mol, equals the calorimetrically measured enthalpy of one G3T quadruplex, −28 kcal/mol (21). Moreover, the *E*_off_ value, 43 kcal/mol, equals the unfolding enthalpy of the first G3T quadruplex from (G3T)_2_. The absolute value of *E*_on_ is smaller than *E*_off_ because G3T quadruplex formation is accompanied by two stacking interactions between G-quartets, whereas the first quadruplex unfolding from (G3T)_2_ is accompanied by breaking of three stacking interactions. Thus, Arrhenius analysis suggests a two-state transition with folding and unfolding of the first G3T quadruplex as the rate-limiting step (Fig. 5 *F*). Although formation of both quadruplexes and DNA duplexes is characterized by a strong negative activation energy consistent with a nucleation zipper mechanism, there is a fundamental difference. For a DNA duplex, the size of the nucleation unit is not strictly defined (i.e., 2–4 bp), whereas for the quadruplex, the nucleation unit is a G3T quadruplex with three G-quartets.

This mechanism shown in Fig. 5 suggests that upon infinitely slow cooling of random-coiled (G3T)_2_, folding of the first G3T quadruplex does not take place until the temperature reaches the phase transition of a G3T monomer (*dashed line*, Fig. 5 *D*). At these temperatures, ∼72°C, folding of G3T is very slow, ∼300 min. However, as soon as the first G3T quadruplex is folded the second unit folds faster since the first quadruplex serves as a platform for stacking the adjacent GGG segments. In other words, although the first G3T domain is stabilized only by hydrogen bonding and cation coordination, the second domain is further stabilized by stacking interactions with the already formed domain. Further cooling is accompanied by acceleration of the process and at 66°C the folding is completed. Upon heating, (G3T)_2_ starts unfolding above 66°C. The unfolding begins from one end and as soon as the first quadruplex is fully unfolded, the second quadruplex melts rapidly since the temperature is significantly above its *T*_m_ of 55°C (see *dashed line in* Fig. 5 *D*).

**Figure 6:**
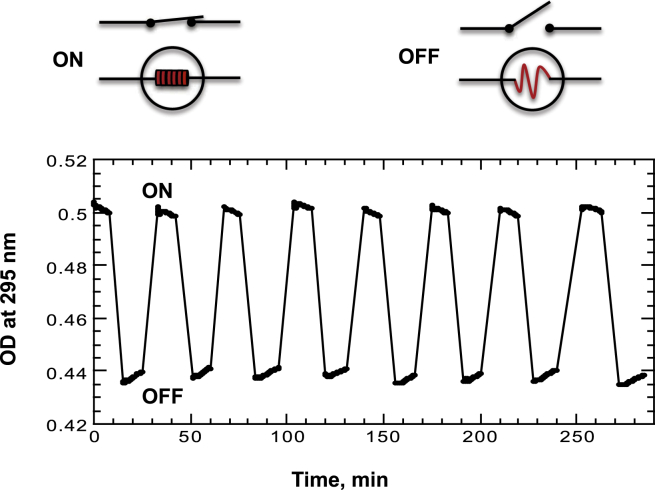
Heat-induced reversible folding and unfolding of a (G3T)_2_ quadruplex at constant temperature, 70°C, monitored by light absorption. Folding and unfolding was achieved by removing the optical cell from the UV spectrometer and incubating the cuvette at 45°C for 3 min to achieve the initial ON state. The cell was returned to the cell holder and following a 2 min equilibration to reach the reaction temperature of 70°C, the UV absorption was recorded for 10–15 min. The OFF state was then achieved by removing the cell and incubating the sample at 95°C for 3 min. The measurement was continued after returning the cell into the cell holder and equilibrating for 2 min to reach the reaction temperature of 70°C. This procedure was repeated a total of eight times. To see this figure in color, go online.

It is well documented that formation of monomolecular quadruplexes could proceed through multistep pathways with intermediate states (i.e., hairpins or triplexes stabilized by G-G interactions) (45, 46, 47). The presence of these types of intermediates can be excluded in the present case based on the following. First, if the slow kinetics, detected around 70°C, is caused by rearrangement of the final quadruplex structure from an intermediate state, then even slower folding rates should be detected at lower temperatures. However, the isothermal experiments show rapid folding at lower temperatures (see Fig. 5 *E*). Moreover, (G3T)_2_ samples prepared by either slow annealing or rapid cooling revealed exactly the same CD profiles and hysteretic loops (Fig. S4). Second, specific single-nucleotide modifications in the middle G3T unit of (G3T)_3_ switch off the hysteresis (Fig. 4). In addition, deletion of even a single guanine from either end of (G3T)_2_ completely eliminates the hysteresis (see Fig. 3). Third, formation of a stable secondary structure other than a G3T quadruplex is highly unlikely under the low ionic strength conditions (0.1 mM KCl) used in these experiments. Fourth, oligo(G3T) hysteretic loops are highly reproducible (Figs. 6 and S2).

Thus, the current study suggests that the hysteresis is due to a highly specific and reproducible two-state transition in which the initial G3T quadruplex formation in tandem G3T sequences serves as a nucleation factor for formation of subsequent quadruplexes. Thus, there is an interesting parallel between oligo(G3T) formation and DNA duplex formation from complementary strands, as both cases the rate-limiting nucleation step is followed by fast zipping.

### (G3T)_n_ hysteresis and its implication

Our study demonstrates that G3T-based tetrahelical DNA exhibits highly reproducible thermal hysteresis. In other words, its state (folded or unfolded) depends not only on the current temperature but also on its history. The quadruplex can thus be considered to be a molecule exhibiting temperature memory. One can take advantage of this phenomenon to design heat-activated DNA switches or reconfigurable DNA structures. To design DNA switches, various external stimuli were previously employed: DNA strands (13, 15), macrocyclic ligands (12), photons (14), and electrochemical signals (16). Only the latter two can be employed for isolated (closed) systems; however, both approaches utilize extra components. The photon-fueled machines use azobenzene (14), which is expensive and requires operation in the dark, whereas the electrochemical switch uses Pb^2+^ (16), which is a highly reactive cation capable of forming covalent bonds with atomic groups of nucleic bases.

Here, we demonstrate the design of a DNA quadruplex switch functioning at constant temperature (for example, at 70°C as shown in Fig. 6) and oscillating between ON (folded) and OFF (unfolded) positions by rapid temperature changes in the absence of any additional chemical component. As shown in Fig. 5, at 70°C both the folding and unfolding of the quadruplexes are very slow; however, the structural transitions occur rapidly at 45°C and 95°C, respectively. Because of the slow kinetics at the intermediate temperature, the quadruplexes can remain in the desired conformation (folded or unfolded) induced by the temperature treatment for a prolonged time period. The feasibility of a heat-activated DNA switch was demonstrated by monitoring the optical properties of (G3T)_2_ measured in a cuvette at 70°C (Fig. 6). Oscillation between ON and OFF positions or folding and unfolding of (G3T)_2_ was achieved by removing the cuvette from the UV spectrometer and incubating in separate water baths equilibrated at 45°C or 95°C, respectively, followed by reequilibration to 70°C and reading the UV absorbance for 10–15 min. This procedure was repeated seven times. Thus the experiment shown in Fig. 6 corresponds to eight sequential switching cycles. During the recording time, a slight increase or decrease is observed corresponding to 5% to 7% of total folding or unfolding. As expected, the experiment demonstrates highly reproducible switching cycles between quadruplex (ON) and random-coil (OFF) states. Because DNA quadruplexes can serve as a nanowires (10, 11), our approach can be employed in designing heat-stimulated DNA transistors.

## Conclusions

In this study, we looked at a monomolecular DNA tetrahelical polymer, oligo(G3T), with highly reproducible hysteretic behavior and experimentally measured kinetic parameters and equilibrium transition profiles. The hysteresis is due to G3T quadruplex domain organization and domain-domain interactions, which dominate the folding energetics of oligo(G3T). As a result, the higher-order oligo(G3T) assemblies unfold at significantly higher temperatures than a single G3T domain. This study reveals that folding and unfolding of the first G3T domain is rate-limiting for both the annealing and melting processes. Although folding requires reaching lower temperatures to stabilize the first G3T domain, unfolding requires significantly higher temperatures to unfold a single domain from the polymer stabilized by domain-domain interactions. Because DNA quadruplexes are good nanowires, G3T-based polymers can be used in designing heat-stimulated DNA nanoswitches or transistors in an isolated (closed) system in the absence of any extra components.

## Author Contributions

B.K. conceived research; B.K., D.G., L.L., N.M., and K.M.F. designed experiments; B.K., D.G., A.R., and L.L. performed experiments and analyzed data; and B.K. and K.M.F. wrote the manuscript.

## Supporting Material

Document S1. Figs. S1–S4

Document S2. Article plus Supporting Material
