# Supplementary material for: Stable Domain Assembly of a Monomolecular DNA Quadruplex: Implications for DNA-Based Nanoswitches
Source: Biophys J. 2016 May 24;110(10):2169–75. doi: 10.1016/j.bpj.2016.04.031 (PMC4880955; doi:10.1016/j.bpj.2016.04.031)
Supplement: Document S1. Figs. S1–S4 [file mmc1.pdf]

Biophysical Journal, Volume *110*

Supplemental Information

Stable Domain Assembly of a Monomolecular DNA Quadruplex: Implications for DNA-Based Nanoswitches

Besik Kankia, David Gvarjaladze, Adam Rabe, Levan Lomidze, Nunu Metreveli, and Karin Musier-Forsyth

Biophysical Journal

Supporting Material

Stable Domain Assembly of a Monomolecular DNA Quadruplex: Implications for DNA-Based Nanoswitches

Besik Kankia,[1,2,*] David Gvarjaladze,[2] Adam Rabe,[1] Levan Lomidze,[2] Nunu Metreveli,[2] and Karin Musier-Forsyth[1]

[1]Department of Chemistry and Biochemistry, The Ohio State University, Columbus, Ohio; and [2]Institute of Biophysics, Ilia State University, Tbilisi, Republic of Georgia

*Correspondence: kankia.1@osu.edu

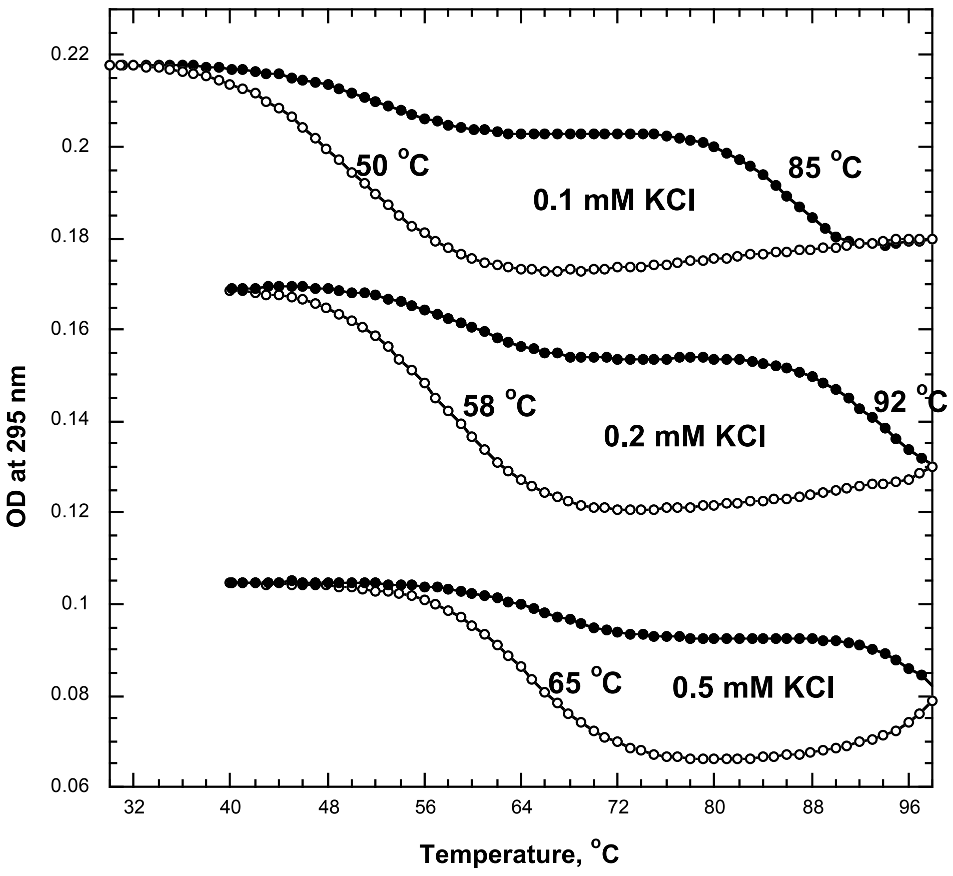

Figure S1. UV melting curves of $(G3T)_2$ (without PAGE purification) in the presence of 0.1, 0.2 and 0.5 mM KCl measured using a 1 ºC/min temperature gradient. Increase in K^+ concentration is accompanied by shift of the hysteresis loops to higher temperatures.

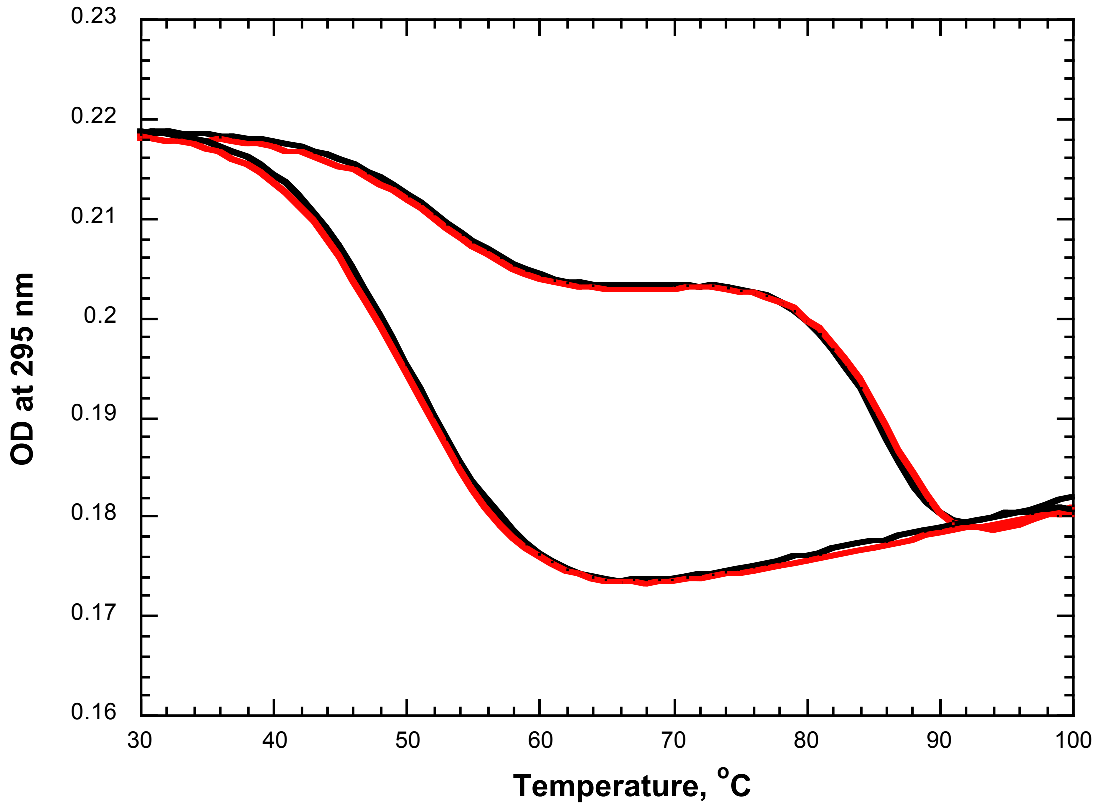

Figure S2. Reproducibility of $(G3T)_2$ (without PAGE purification) UV melting/annealing curves in the presence of 0.1 mM KCl measured using a 1 ºC/min temperature gradient. Red and black curves correspond to independent experiments.

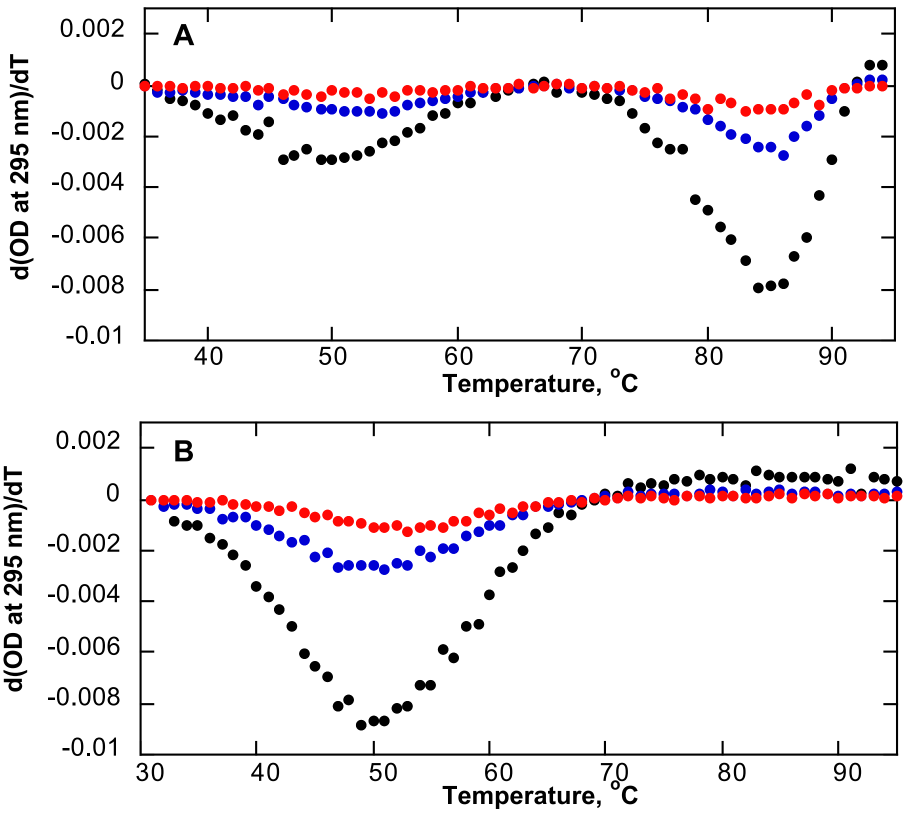

Figure S3. UV unfolding (A) and refolding (B) curves of $(G3T)_2$ (without PAGE purification) at 1 μM (red), 3 μM (blue) and 10 μM (black) strand concentration in the presence of 0.1 mM KCl measured using a 1 ºC/min temperature gradient.

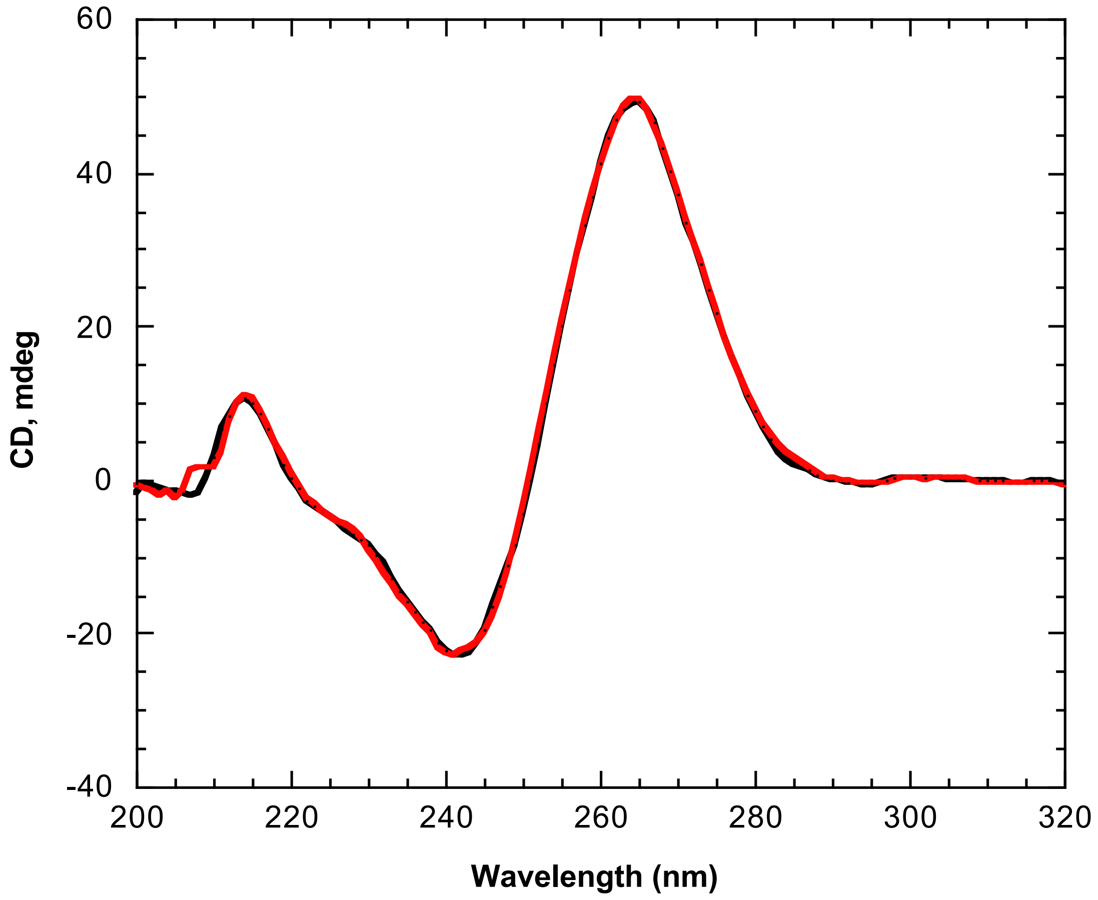

Figure S4. CD spectra of $(G3T)_2$ at 20 °C, in the presence of 0.1 mM KCl, prepared either by rapid cooling (black) or slow annealing (red) revealed similar CD profiles corresponding to all-parallel quadruplexes.
